# Dopamine Modulates Serotonin Innervation in the *Drosophila* Brain

**DOI:** 10.3389/fnsys.2017.00076

**Published:** 2017-10-16

**Authors:** Janna Niens, Fabienne Reh, Büşra Çoban, Karol Cichewicz, Julia Eckardt, Yi-Ting Liu, Jay Hirsh, Thomas D. Riemensperger

**Affiliations:** ^1^Molecular Neurobiology of Behavior, Johann-Friedrich-Blumenbach-Institute for Zoology and Anthropology, University of Göttingen, Göttingen, Germany; ^2^Department of Biology, University of Virginia, Charlottesville, VA, United States

**Keywords:** Parkinson’s disease, dopamine, serotonin, *Drosophila melanogaster*, neuroanatomy, plasticity

## Abstract

Parkinson’s disease (PD) results from a progressive degeneration of the dopaminergic nigrostriatal system leading to a decline in movement control, with resting tremor, rigidity and postural instability. Several aspects of PD can be modeled in the fruit fly, *Drosophila melanogaster*, including α-synuclein-induced degeneration of dopaminergic neurons, or dopamine (DA) loss by genetic elimination of neural DA synthesis. Defective behaviors in this latter model can be ameliorated by feeding the DA precursor L-DOPA, analogous to the treatment paradigm for PD. Secondary complication from L-DOPA treatment in PD patients are associated with ectopic synthesis of DA in serotonin (5-HT)-releasing neurons, leading to DA/5-HT imbalance. Here we examined the neuro-anatomical adaptations resulting from imbalanced DA/5-HT signaling in *Drosophila* mutants lacking neural DA. We find that, similar to rodent models of PD, lack of DA leads to increased 5-HT levels and arborizations in specific brain regions. Conversely, increased DA levels by L-DOPA feeding leads to reduced connectivity of 5-HT neurons to their target neurons in the mushroom body (MB). The observed alterations of 5-HT neuron plasticity indicate that loss of DA signaling is not solely responsible for the behavioral disorders observed in *Drosophila* models of PD, but rather a combination of the latter with alterations of 5-HT circuitry.

## Introduction

Parkinson’s Disease (PD) is a progressive degeneration of the dopaminergic nigrostriatal system leading to a decline in movement control associated with resting tremor, rigidity and postural instability. A small minority of PD cases are linked to pathogenic gene mutations responsible for the development of the disease. However, studies of rare Mendelian forms of PD allowed for the identification of more than 20 PD genes and variants that are implicated in its development (Rousseaux et al., 2017). Whereas, about 10–15 percent of Parkinson’s patients are thought to suffer from a genetic form of this dystonic movement disorder, most patients suffer from a sporadic form of PD most likely resulting from a combination of environmental factors and undefined individual genetic susceptibility (Obeso et al., 2014; Ascherio and Schwarzschild, 2016). Whether the underlying causes act separately or converge into common pathways remains to be resolved. Both sporadic and hereditary pathogenic events lead to the disease that affects the survival of dopamine (DA) producing neurons in vulnerable brain areas such as the substantia nigra (Westerlund et al., 2010).

Studies in 6-hydroxydopamine (6-OHDA)-treated rats displaying nigrostriatal DA lesions indicate that lack of DA signaling promotes the growth of 5-HT neurons in the striatum (Zhou et al., 1991). The observed hyper-innervation of serotoninergic neurons that can be induced by DA neuron denervation imply that it is not exclusively the loss of DA signaling that is responsible for the development of the PD-triggered disorders, but rather a combination of the latter with alterations of 5-HT circuitry.

The powerful genetic tools available in *Drosophila* make it an excellent model system to study the cellular mechanisms underlying neurodegenerative diseases (Feany and Bender, 2000; Marsh and Thompson, 2006; Lu, 2009; Dehay and Bezard, 2011; Riemensperger et al., 2013; Vanhauwaert and Verstreken, 2015; West et al., 2015; Hewitt and Whitworth, 2017). In recent years, *Drosophila* has proven to be a valuable model system for dopaminergic neurodegeneration under conditions mimicking PD. For instance, ectopic expression of a mutated form of human α-synuclein, α-synA30P, in *Drosophila melanogaster* models the dopaminergic neurodegeneration seen in vertebrates (Feany and Bender, 2000), and the presence of α-synA30P in subsets of the protocerebral anterior medial protocerebrum (AMP) DA neurons leads to gradual loss of their projections onto target neuropils (Riemensperger et al., 2013).

Fruit flies mutant for the enzyme tyrosine hydroxylase (TH), which are unable to produce DA in the central nervous system (CNS), show a variety of behavioral deficits (Hirsh et al., 2010; Riemensperger et al., 2011; Cichewicz et al., 2016), demonstrating the crucial role of DA in the control of diverse behaviors (Friggi-Grelin et al., 2003; Schwaerzel et al., 2003; Andretic et al., 2005; Kume et al., 2005; Ganguly-Fitzgerald et al., 2006; Liu et al., 2008; Lebestky et al., 2009; Riemensperger et al., 2011; Ueno et al., 2012; Owald and Waddell, 2015; Nall et al., 2016; Fiala and Riemensperger, 2017). However, to understand the development of the wide range of behavioral disorders deriving from a DA dysregulation, it is crucial to understand how neuronal circuits react to the loss of DA signaling in the CNS. Here, we have investigated how long-term or acute changes in DA-signaling affect serotonin-neuron plasticity in *Drosophila*.

## Materials and Methods

### *Drosophila* Strains

Either Canton S (CS) or *w*^1118^, back-crossed for seven generations to CS, were used as wild-type control flies. Brain DA-deficient *Drosophila* (*d*TH def.) were *DTH*^*FS*+/−^ BAC *ple*^2^ and rescue controls (*d*TH resc.) were DTH BAC *ple*^2^ (as per Cichewicz et al., 2016). Animals used for immunohistochemistry were 3–5 days post eclosion. For reconstitution of splitGFP experiments TrH-Gal4 flies (Cassar et al., 2015) were crossed with flies carrying a combination of UAS:splitGFP1-10 (Pech et al., 2013), DsRed (Riemensperger et al., 2005) and splitGFP11 under the control of the mb247 promotor (Pech et al., 2013). Flies were raised under standard conditions at a 12:12 h light-dark schedule at 25°C and 60% relative humidity.

### Immunohistochemistry

If not otherwise indicated, brains of 3- to 5-day old female flies were dissected in ice-cold Ringer’s solution containing 5 mM HEPES-NaOH (pH = 7.4), 130 mM NaCl, 5 mM KCl, 2 mM MgCl_2_, 2 mM CaCl_2_, and 36 mM sucrose, and fixed for 2 h on ice in 4% paraformaldehyde dissolved in phosphate-buffered saline (PBS), and subsequently washed three times in PBS containing 0.6% Triton X-100. Samples were incubated overnight at 4°C in PBT containing 2% bovine serum albumin (BSA). If not otherwise indicated, the samples were subsequently incubated with mouse anti-TH (Immunostar, dilution 1:100) with rabbit anti-5-HT (Sigma-Aldrich, dilution 1:500) or for corroboration of the results, with other 5-HT antibody (Supplementary Figure S1; rat anti-5HT, Merck, 1:100) diluted in block solution at 18°C for at least 6 h. After washing the samples at least three times for 20 min each with PBS containing 0.6% Triton X-100, the brains were incubated at 4°C overnight in secondary antibodies diluted in PBS containing 0.6% Triton X-100. Secondary antibodies were anti-mouse Alexa 488-conjugated (Invitrogen, 1:300) or anti-mouse Cy3-conjugated (Jackson, 1:300). Samples were then washed again three times in PBS containing 0.6% Triton X-100, incubated for at least 6 h in PBS, and mounted in Vectashield (Vector Laboratories). Images were taken using a Leica SP8 confocal microscope equipped with a Leica Apochromat 20×/0.7 water immersion objective. The brains were scanned at 1.0 μm steps in the z-axis with a resolution of 1.0 μm/pixel. Images were analyzed using ImageJ. To determine fluorescence intensities, scans were transformed to z-projections and analyzed as described in Neckameyer and Bhatt (2012).

### L-DOPA Treatment

Flies were incubated for 5–10 days at 25°C on standard fly food containing 1 mg/mL L-DOPA (D9628, Sigma-Aldrich). Test and control flies were transferred to fresh food of the according regimen every second day.

## Results

### Dopamine- and Serotonin-Producing Neurons Joint and Complementary Innervation Patterns in the Brain

**Figure 1:**
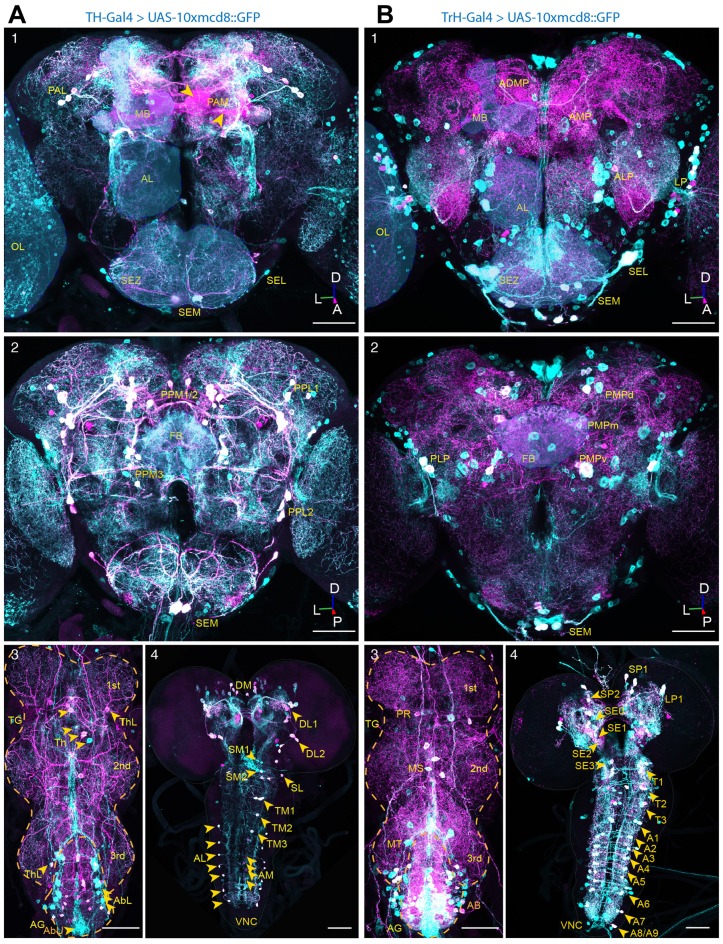
Dopaminergic and serotoninergic neurons of the adult *Drosophila* central brain.** (A)** *In situ* co-immunostainings with anti-GFP (*green*) and anti-tyrosine hydroxylase (TH; *magenta*) antibodies in whole-mount nervous tissues of *TH-Gal4 >10xUAS-mCD8<GFP* flies. Co-localizations merge both colors in *white*, showing driver-targeted somata of the subesophageal medial (SEM), lateral (SEL), the protocerebral medial (PAM) and lateral (PAL) clusters and projections in anterior **(1)** and the posterior lateral (PPL1, PPL2) and medial clusters (PPM1/2, PPM3) in the posterior **(2)** brain, as well as the lateral (ThL) and medial cell cluster (Th) in the thoracic ganglion (TG) and the lateral (AbL) and medial (AbU) situated clusters in the abdominal ganglion (AG) **(3)** of adult wild-type *Drosophila*. In the central nervous system (CNS) of LIII-Larvae **(4)** the dopaminergic system consists of one dorso-medially (DM), two dorso-laterally (DL1, DL2) within the hemispheres, three clusters in the medial (SM0, SM1, SM2) and one cluster in lateral (SL) subesophageal zone (SEZ), three medially situated cluster in the thoracic (TM1, TM2, TM3) clusters and an array of neurons in the lateral (AL) and medial (AM) part of the abdominal part of the ventral nerve cord (VNC). **(B)** *In situ* co-immunostainings with anti-GFP (*green*) and anti-5-HT (*magenta*) antibodies in whole-mount nervous tissues of *TrH-Gal4>10xUAS-mCD8<GFP* flies, showing the clusters in the lateral lateral protocerebrum (LP), the anterior medial protocerebrum (AMP), anterior lateral protocerebrum (ALP) and the anterior dorso-medial protocerebrum (ADMP) and the medially (SEM) and laterally (SEL) situated clusters in the subesophagealsubesophageal zone (SEZ) in the anterior **(1)** and the clusters situated in the dorsal (PMPd), medial (PMPm) and ventral posterior protocerebrum as well as the dorsally situated clusters in the medial (SEM) and lateral (SEL) subesophageal ganglion **(2)** and the clusters in the por-, (PR), meso- (MS) and meta- (MT) thoracic neuromere and the AG (AB) **(3)** of an adult fly. The CNS of a LIII-Larva **(4)** of three clusters in the supresophageal ganglion (SP0, SP1, SP2), one in the LP1 and four cell clusters in the SEZ (SE0, SE1, SE2, SE3). The VNC contains three clusters of 5-HT producing neurons in the thoracic (T1, T2, T3) and an array of nine symmetrically organized clusters in the abdominal neuromere (A1–A9). Overlay in white correspond to driver-targeted serotoninergic cell bodies (MB, mushroom body; FB, fan-shaped body; OL, optic lobe; SEZ, subesophageal zone; AL, antennal lobe; TG, thoracic ganglion; AG, abdominal ganglion; D, *dorsal*; L, *lateral*; P, *posterior*; A, *anterior*). Scale bars: 50 μm.

To visualize the differential innervation of serotoninergic and dopaminergic neurons in the *Drosophila* central brain, we stained with antisera to 5-HT, and to TH, in conjunction with TH-Gal4- (Friggi-Grelin et al., 2003) and Trh-Gal4- (Cassar et al., 2015) driven GFP in DA and 5-HT neurons, respectively (Figures 1A,B). In agreement with previously published observations (Monastirioti, 1999), the anterior dopaminergic system of the adult brain mainly consists of three neuronal clusters, including neurons situated laterally in the anterior protocerebrum (PAL), a small group of neurons located in the lateral and medial parts of the subesophagal zone (SEZ) and about 100 neurons in the medial protocerebrum (PAM). In several aspects, the neurons of the PAM cluster differ from the other clusters of the DA system. PAM cluster neurons are the last born DA producing neurons and develop only during pupation. They have much smaller somata and they are the only DA producing neurons of the CNS that are for most (~85%) of them not included in the expression pattern of the TH-Gal4 line, indicating that these neurons also differ at the level of gene regulation. Being the only neurons of the anterior DA system innervating the mushroom body (MB), these neurons send their projections towards the tips of the γ-lobes, the β’-lobes and the shaft of the β-lobes (Figure 1A1, yellow arrow heads; see also Pech et al., 2013). The posterior DA system of the adult brain consists of the PPL1, PPL2, PPM1/2, PPM3, a group of small neurons located lateral to the SEZ, and a group of large neurons in the medial part of the SEZ. The neurons of the posterior cluster, positioned lateral to the MB calyx (PPL1), densely innervate the heel and the vertical lobes of the MB. The cluster located between the CNS and the optic lobes (OL) innervate the lobula plate at the ipsilateral side and send their projections to the lobula plate of the contralateral OL (Figure 1A2).

The dopaminergic system of the adult thoracic nerve cord consists of dense innervations deriving partially from the CNS and projecting to all three segments, as well as from groups of dopaminergic neurons positioned laterally (Figure 1A3, ThL) or medially between the first and second (Figure 1A3, Th) and between the second and the third segment, as well as between the third thoracal segment and the abdominal ganglion (AG; Figure 1A3, ThL). The dopaminergic neurons of the AG are positioned laterally to the ganglion and send their projection to the tip of the AG (Figure 1A3, AbL), where a second group of DA producing neuron in the AG is positioned (Figure 1A3, AbU). The central DA system of third-instar (LIII) larvae is largely comparable to the one of adult flies, with the exception of the PAM cluster that consists of only four neurons per hemisphere in LIII-larvae and counts about 100 neurons in adults. Despite the much simpler anatomy of the larval DA system, similar functions of these neurons were described for adults and larvae (Rohwedder et al., 2016). In the larval ventral ganglion, lateral DA neurons send long projections to the neuropil where they form lateral, longitudinal bundles and from whence they project towards the medial part of the ventral ganglion, whereas medially positioned DA neurons with short projections form a medial, longitudinal bundle, projecting to the lateral parts of the ventral ganglion (Figure 1A4).

**Figure 2:**
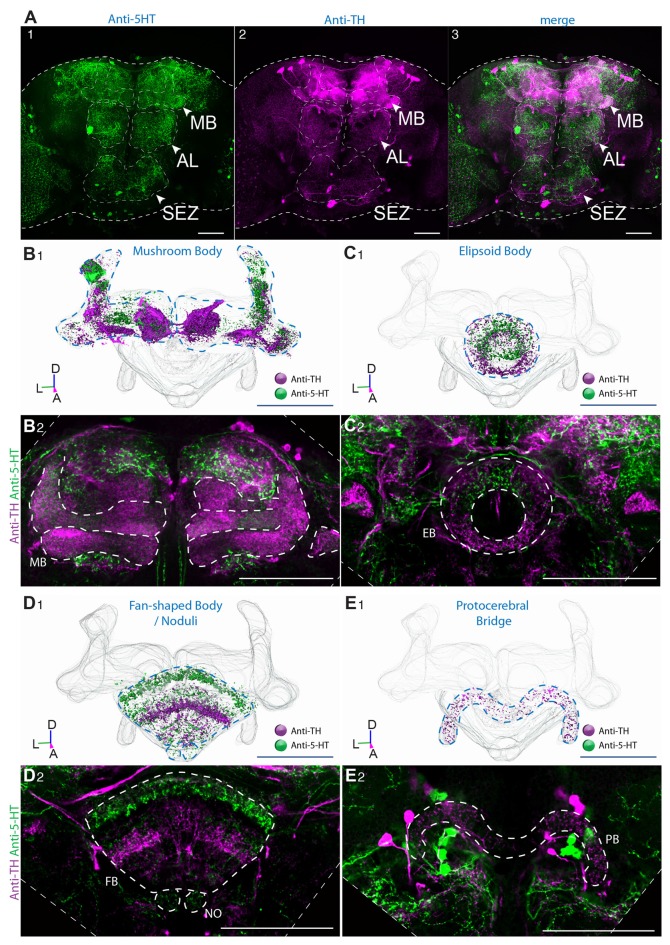
Innervation pattern of dopaminergic and serotoninergic neurons in the adult wild-type *Drosophila* central brain. **(A)** Anti-5-HT **(1)** and anti-TH **(2)** immunoreactive neurons show to some degree complementary innervation patterns in the *Drosophila* brain. 5-HT-producing neurons strongly innervate the AL **(A)** and only weakly the MBs **(B1,2)**. TH-positive neurons innervate only the outer rim of the AL **(A2,3)**, but strongly the mushroom body (MB) **(B1,2)**. The tips of the α’ lobes are innervated by both populations of aminergic neurons **(B1–2)**. Innervation pattern of 5-HT neurons and dopamine (DA) neurons in the ellipsoid body (**C1,2**, EB) and the fan-shaped body (**D1–2**, FB). 5-HT-producing neurons innervate strongly the inner rim of the EB **(C1,2)** and the dorsal part of the FB **(D1,2)**. DA-producing neurons innervate strongly the outer rim of the EB **(C1,2)** and the ventral part of the FB (**D1,2**). The protocerebral bridge (PB) is innervated by DA, but by 5-HT producing neurons **(E1,2)**. 3D reconstruction of the MB **(B1)**, EB **(C1)**, the FB **(D1)** and the PB **(E1)** with DA and 5-HT IR indicated in different colors (D, *dorsal*; L, *lateral*; P, *posterior*; A, *anterior*). Scale bars: 50 μm.

The CNS of adult *Drosophila* is densely innervated by ~90 5-HT producing interneurons that can be subdivided in 10 clusters (Vallés and White, 1988; Sitaraman et al., 2008; Alekseyenko et al., 2010; Sadaf et al., 2012; Pech et al., 2013; Pooryasin and Fiala, 2015; Figures 1B1,B2). Out of these ~90 5-HT producing neurons about 80 neurons can be targeted by TrH-Gal4 (Sitaraman et al., 2008; Cassar et al., 2015) that however drives ectopic expression in nearly 170 non-serotoninergic neurons (Figure 1B; see also Pooryasin and Fiala, 2015). Similar to vertebrates, where neuropils that are innervated by DA producing neurons are also innervated by 5-HT neurons (Niederkofler et al., 2015), with the exceptions of the antennal lobes (AL) that are innervated by 5-HT producing neurons but only sparsely at the outer rim by DA neurons (Figures 2A1–3) and the protocerebral bridge (PB) that is innervated by DA neurons but not by 5-HT producing neurons (Figure 2E1), both aminergic systems innervate the same target regions, but differ in their density on local innervation patterns. At the anterior side, the serotoninergic system can be subdivided in a medial (AMP) and a dorsomedial (ADMP—anterior dorso medial protocerebrum) cluster and a small group of neurons positioned in the anterior lateral protocerebrum (ALP) and a large of group of neurons positioned between the OL and the CNS in the lateral protocerebrum (LP). The SEZ is innervated at the anterior side by a cluster of large lateral neurons (SEL) and a group of small medial neurons (SEM), whereas at the dorsal side a group of small neurons is positioned laterally, and a group of three neurons is located at the medial part (SEM) in direct vicinity to a group of large DA producing neurons (Pooryasin and Fiala, 2015; Figure 1B1). At the posterior side, the 5-HT producing neurons can be classified in a medially positioned cluster that can be further subdivided in the posterior medial dorsal (PMPd), posterior medial (PMPm) and posterior ventral cluster (PMPv). In the LP a group of two neurons with strong 5-HT immunoreactivity (IR) forms the posterior lateral protocerebral cluster (LP; Pooryasin and Fiala, 2015; Figure 1B2).

The thoracic ganglion (TG) is strongly innervated by 5-HT neurons partially deriving from the CNS and projecting to all three segments as well as from groups of neurons positioned medially between the first and second (Figure 1B3, PR) and between the second and the third segment Figure 1B3, MS). The 5-HT neurons of the AG are positioned laterally and medially in the ganglion sending their projection to the tip of the AG (Figure 1B3, AB, MT). The 5-HT system of LIII larvae has been described in detail (Vallés and White, 1988; Huser et al., 2012) and consists mainly of four clusters in the brain hemispheres, and four clusters in the SEZ. In the larval ventral ganglion 5-HT neurons with short projections innervate the neuropil at the ipsilateral side and 5-HT neurons with long projections innervate the contralateral side or both sides of the ganglion (Figure 1B4; Huser et al., 2012).

Both the AL (Figures 2A1–3) and MB (Figures 2A1–3,B1,B2) are contacted by fine 5-HT terminals. In agreement with Vallés and White (1988), who used a different antibody, we find that in the CC, a structure composed of the ellipsoid body (Figures 2B1,B2, EB), the fan-shaped body (FB; Figures 2C1,C2, FB), the noduli (Figures 2D1,D2, NO) and the PB (Figures 2E1,E2, PB), 5-HT-producing neurons predominantly send their projections to the inner rim of the EB (Figures 2E1,E2) and to the superior arch of the FB (Figures 2D1,D2), but only sparsely into the noduli (Figures 2D1,D2). With the exception of the AL, which is only sparsely innervated by TH immunoreactive neurons at the outer rim and the PB that is exclusively innervated by DA producing neurons (Figures 2E1,E2), DA and 5-HT producing neurons project to the same neuropils, but differ in their exact innervation characteristics within their target neuropils. Whereas the MB is sparsely, but homogeneously innervated by the 5-HT producing dorsal pair medial (DPM) neuron, DA producing neurons group in different sub-populations that form dense and spatially restricted innervations onto characteristic sub-regions within the MB lobes (Figures 2B1,B2; see also Pech et al., 2013), consistent with immunocytochemical pattern observed with an antibody to DA (Cichewicz et al., 2016). The terminal projections of DA-producing neurons into the CC are mainly complementary to those of 5-HT producing neurons. TH immunoreactive neurons innervate densely the outer rim of the EB (Figures 2C1,C2) and the medial parts of the FB (Figures 2D1,D2), whereas 5-HT projections are concentrated to the inner and anterior part of the EB (Figures 2C1,C2) and the dorsal FB (Figures 2D1,D2).

In summary, we find in the logic underlying the innervation pattern of the DA and 5-HT system similarities to the innervations characteristics of aminergic systems in vertebrates. DA- and 5-HT-producing neurons innervate large portions of the CNS, with largely overlapping target neuropils. With the exception of the AL and the PB, both 5-HT- and TH-immunoreactive neurons send their projections to the same neuropils, but differ mainly in the densities of their innervation within sub-region of the particular neuropil.

### DA-Deficient Flies Show Increased 5-HT Immunoreactivity (IR) in the Posterior Lateral Protocerebral Neurons

**Figure 3:**
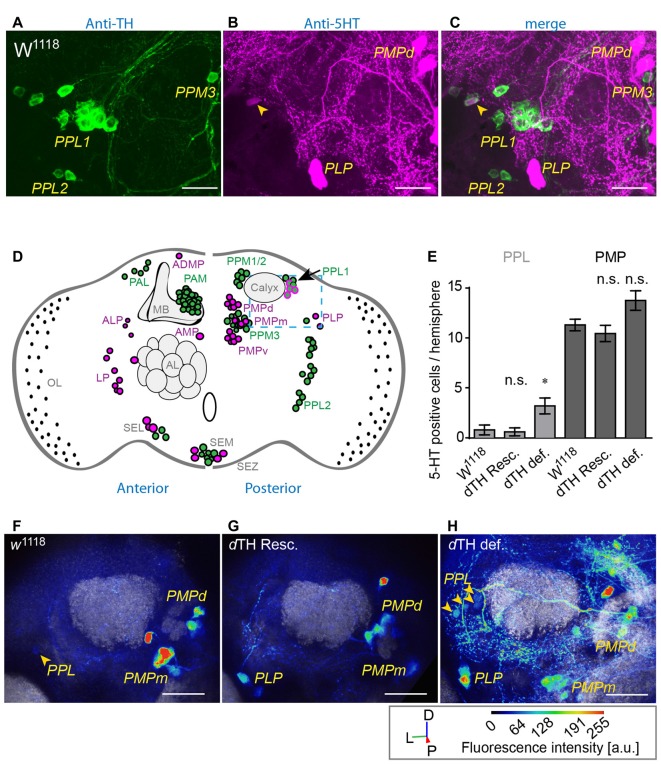
DA-deficient flies show increased 5-HT immunoreactivity (IR) in TH-producing neurons of the posterior lateral protocerebrum (PLP).** (A)** TH- (green) and **(B)** 5-HT- immune reactive neurons (green) in the posterior lateral protocerebrum (PPL) and the posterior medial protocerebrum (PMP) in *w*^1118^. Merge is shown in **(C)**. Scale bars: 10 μm. *W*^1118^ control flies show a small number of faintly 5-HT-immunoreactive neurons (magenta) among **(A)** DA-producing PPL1 neurons (**C**, arrow heads). **(D)** Schematic overview of the TH (green) and 5-HT (magenta) immune reactive neurons in the adult *Drosophila* brain. In the PPL (blue dashed line) *w*^1118^ control flies show a small number of 5-HT-immunoreactive neurons (magenta) among DA-producing neurons (green) in the PPL1 cluster (arrow). **(E)** DA-deficient flies show increased numbers of 5-HT-producing neurons in the PPL when compared to *w*^1118^ controls and dTH-rescue flies (Dunn’s multiple comparison test against *w*^1118^, *n* > 5). **(F)** 5-HT immune reactive neurons (green) in the PPL and the PMP in *w*^1118^, genomic *d*TH rescue (**G**, *d*TH Resc.) and DA-deficient flies (**H**, *d*TH def., arrow head) (D, *dorsal*; L, *lateral*; P, *posterior*) Scale bars: 20 μm. n.s.: *p* > 0.05; *p < 0.05.

**Figure 4:**
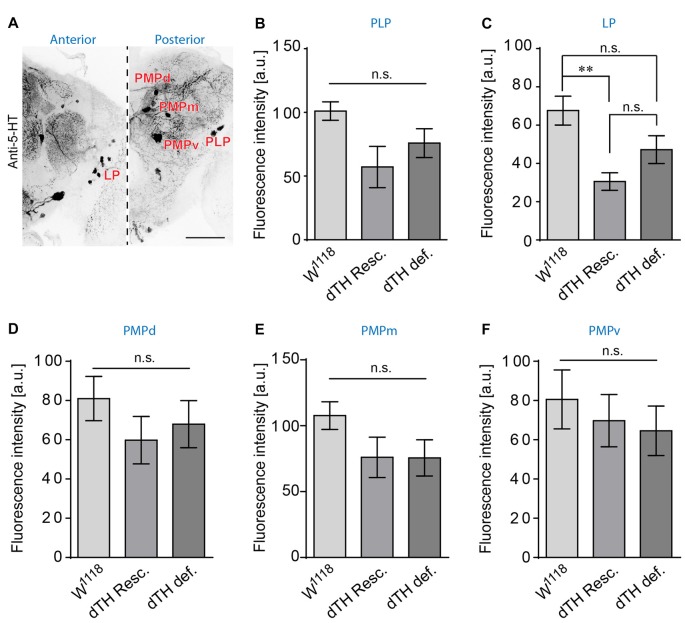
DA deficiency does not increase 5-HT IR in somata of 5-HT-producing neurons.** (A)** 5-HT immunoreactive neurons in the anterior (left, LP) and posterior medial (right, PMPd, PMPm, PMPv) and PLP in *w*^1118^. Scale bars: 50 μm. **(B–F)** DA-deficient flies do not show increased 5-HT somatic IR when compared to *w*^1118^ or genomic *d*TH-rescue flies (ANOVA with Bonferroni correction, *n* < 6). n.s.: *p* > 0.05; **p < 0.01.

To analyze the impact of DA loss on the serotoninergic system, we compared the anatomy of flies lacking *d*TH in the CNS, compared to *d*TH-rescue flies and *w*^1118^ control flies. We find that the overall appearance of the serotoninergic system of flies lacking DA is comparable to that of *d*TH rescue flies or *w*^1118^ controls. However, brains lacking genomic *d*TH show an increased number of 5-HT immunoreactive somata within the posterior lateral protocerebrum (PLP; Figures 3A–H, PPL). In control *w*^1118^ 5-HT positive neurons in the PLP were described as positioned ventrally to the calyces (Figures 3A–D), near the OL (Figure 3D; Vallés and White, 1988; Monastirioti, 1999; Sitaraman et al., 2008; Cassar et al., 2015; Pooryasin and Fiala, 2015). Giang et al. (2011) further have identified an additional group neurons with faint IR against the 5-HT transporter positioned laterally to the calyces similarly positioned then the PPL1 dopaminergic neurons. In wild-type brains, a small number of TH IR neurons within the PPL1 cluster show very faint 5-HT IR (Figures 3A–C). However, in DA-deficient brains, we find an increased 5-HT IR of neurons juxta-calycal in the PLP (Figure 3H, PPL) at the position of the PPL1 DA producing neurons (Nässel and Elekes, 1992; Monastirioti, 1999; Riemensperger et al., 2013). This increased IR we could verify with two different antibodies against 5-HT (Supplementary Figure S1). However, it is not clear whether the increased 5-HT IR in DA deficient flies derives from the same neuronal populations. Other clusters, like the PMP clusters (Figure 3D), did not reveal any changes in number of visible somata of 5-HT IR. To exclude the possibility that the observed increase in number of these PPL1-like 5-HT neurons observed in DA-deficient brains does not derive from globally increased 5-HT production, we quantified 5-HT IR between DA-deficient flies, rescue flies and *w*^1118^ control flies in the lateral protocerebrum (LP), PLP, PMPd, PMPm and PMPv 5-HT neuron cluster. With the exception of the LP cluster that showed decreased 5-HT IR in *d*TH-rescue when compared to *w*^1118^ flies, all three strains showed comparable levels of 5-HT IR in the somata of the PMP neurons (Figures 4A–F). It thus appears that increased 5-HT synthesis in the 5-HT/DA PPL1 neurons in the PLP is a selective response occurring in this neuronal cluster that has the capability of increased re-uptake or synthesis of either or both transmitters.

### 5-HT Neurons Projecting to the MB Show Altered Innervation Densities in DA-Deficient Flies

**Figure 5:**
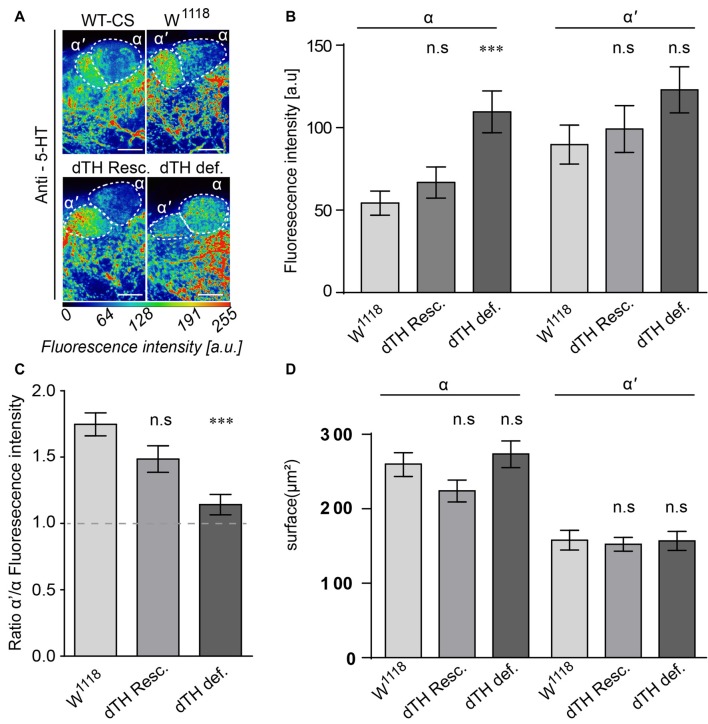
5-HT neurons projecting to the MB show altered innervation densities in DA-deficient flies.** (A)** Innervation patterns of 5-HT neurons to the α/α’-lobe of the MB. Under wild-type conditions, 5-HT producing neurons innervate strongly the tips of the α’ but only faintly the α lobes. In DA-deficient flies, 5-HT innervations to the α lobes are markedly increased. Scale bar: 10 μm **(B)** Fluorescence intensity analysis of the 5-HT IR in α– and α’-lobes. 5-HT innervations to the α-lobes are increased in flies lacking neuronal TH when compared to control flies. **(C)** Ratio of 5-HT innervation density between α’- and α-lobes. Under control conditions, 5-HT neurons innervate α’-lobes stronger than the α lobes. In DA-deficient flies, 5-HT-neuron α-lobe innervations are increased and comparable to the α’-lobe innervations. **(D)** The surface of the α/α’-lobes that are targeted by 5-HT neurons is not altered between DA-deficient and control flies (Dunn’s multiple comparison test against *w*^1118^, *n* < 13). n.s.: *p* > 0.05; ***p < 0.001.

In vertebrate models of PD, degeneration of DA neurons leads to modifications in 5-HT neurons (Zhou et al., 1991; Rylander et al., 2010; Zeng et al., 2010; Niederkofler et al., 2015). To determine whether the same holds true for *Drosophila*, we analyzed the innervation pattern of 5-HT neurons onto their target region in the vertical MB lobes. The tips of the vertical α- and α’-lobes are densely innervated by both TH- and 5-HT-IR neurons. Whereas TH-IR neurons innervate both regions with similar intensities (Figures 2E1,E2), in wild-type brains, 5-HT neurons mainly send projections towards the α’-lobes, but only faintly to the α-lobe (Figures 2D1,D2, 5A). However, in the DA-deficient brains, there is a strong increase in IR for 5-HT in the α-lobes, but not in the α’-lobes (Figure 5B). This increase in 5-HT-immunoreactive projections onto the α-lobes results in a shift in the proportion of 5-HT positive projections between the two lobe structures (Figure 5C). However, it does not affect the overall size in terms of area surface of the innervated neuropils (Figure 5D).

### Long-Term L-DOPA Treatment Alters 5-HT Projections to their Target Neuropils in the CNS

**Figure 6:**
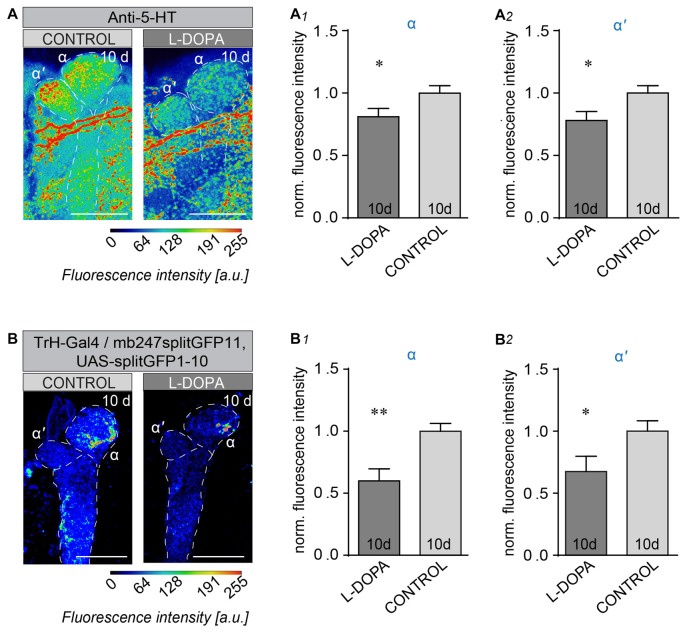
Long-term L-DOPA treatment alters 5-HT neuron innervation to their MB target regions. **(A)** Innervation patterns of 5-HT neurons to the α/α’-lobe of the MB. Projection of 5-HT producing neurons onto α/α’ lobes of the MB under control conditions and after 10 days L-DOPA administration. Fluorescence intensity analysis of the 5-HT IR in α-lobes **(A1)** and α’-lobes (**A2**; unpaired students *T*-test, *n* < 15). **(B)** Reconstituted splitGFP between TrH-positive 5-HT producing neurons and the MB vertical lobes in wild-type flies under control conditions (left) and after 10-day L-DOPA treatment (right). Fluorescence intensities are indicated by false colors. Fluorescence intensity analysis of the reconstituted splitGFP signal in the tips of α **(B1)** and α’ lobes **(B2)**. 5-HT innervations are strongly reduced by L-DOPA treatment compared to non-treated control flies (Unpaired students *T*-test, *n* < 11). Scale bars: 20 μm. n.s.: *p* > 0.05; *p < 0.05; **p < 0.01.

We next asked whether enhanced DA levels, attained by feeding wild-type flies L-DOPA, would affect the serotoninergic system in the opposite way than lack of CNS DA. To this end we analyzed the IR of 5-HT projections onto their target region in the vertical MB lobes after 10 days L-DOPA treatment. 5-HT IR was significantly decreased in both, α- (Figures 6A,A1), and α’-lobes (Figures 6A,A2) after L-DOPA treatment when compared to control flies. To determine whether the observed reduction in 5-HT IR was caused by altered 5-HT biosynthesis or altered plasticity we used the splitGFP technique (Gordon and Scott, 2009; Pech et al., 2013) to visualize alteration in connectivity between 5-HT producing neurons and the MB vertical lobes. Adult flies expressing one part of the splitGFP in 5-TH neurons and the counterpart in MB Kenyon neurons were fed for 10 days with L-DOPA, and monitored for changes in 5-HT GFP fluorescence. Whereas the general pattern of 5-HT IR appeared largely unchanged and comparable to what has been published before (Pech et al., 2013), we found that certain 5-HT projections were significantly decreased (Figure 6B), as can be seen for the intensity of the signal of reconstituted splitGFP between 5-HT terminals and MB lobes at the tip of the α- (Figures 6B,B1) and α’-lobes (Figures 6B,B2). Thus, 5-HT neurons sharing the same target regions than DA neurons respond with diminished projections with enhanced DA, and enhanced 5-HT IR in the absence of DA. These observations provide evidence of competitive interactions between 5-HT and DA in the *Drosophila* brain.

## Discussion

Here we investigated the effects of altered DA signaling on the 5-HT circuitry in the CNS of adult fruit flies. As in vertebrates (Niederkofler et al., 2015), the DA and 5-HT neurons of *Drosophila* send their projections to many brain areas. Most neuropils that are innervated by TH-immunoreactive neurons are also innervated by 5-HT-producing neurons. Whereas the AL is mainly innervated by 5-HT neurons and only faintly at the outer rim by TH immunoreactive neurons, the PB appears to be innervated exclusively be TH-positive neurons, but not by 5-HT. These innervations resemble to some extend the situation in the vertebrate brain where projections of DA-producing neurons are for the most part accompanied by 5-HT producing neurons (Niederkofler et al., 2015).

We found a previously underappreciated set of neurons that co-express DA and 5-HT in the adult fly brain. These DA neurons, comprising 1–2 neurons of the PPL1 cluster, function in conveying an aversive stimulus when stimulated (Masek et al., 2015). In a normal brain, the PPL1 cluster is positioned in a region that is largely devoid of other 5-HT-immunoreactive cell bodies, but IR against the 5-HT transporter in this region has been described beforehand (Giang et al., 2011). However, we find that some of the PPL1 neurons express low levels of 5-HT IR in brains with normal DA synthesis. This 5-HT IR increases significantly in brains lacking DA, both in the cell bodies and in the terminal regions of these neurons, and, therefore, may reflect a potentially compensatory response to DA loss. Whether all of the 5-HT-positive neurons detected indeed correspond to TH-positive PPL1 neurons under wild type conditions remains unknown at the current state. Further investigations on the nature of these neurons and on the mechanisms of how the presence of *d*TH or DA may potentially affect the cell fate of other neurons is needed.

We also found enhanced 5-HT IR in DA-deficient brains in the terminal regions of the PPL1 neurons, in the MB α-lobe. This region is strongly innervated by both DPM, 5-HT and the DA PPL1 neurons. These changes may possibly be explained by 5-HT being now expressed or taken up more strongly in these PPL1 neurons or, as observed in vertebrate models for PD, where denervation of DA neurons was found to potentiate 5-HT IR at neuronal terminals (Zhou et al., 1991; Rylander et al., 2010; Zeng et al., 2010; Niederkofler et al., 2015), derive from altered 5-HT plasticity.

DA deficiency has consequences on a broad variety of behaviors in *Drosophila* (Hirsh et al., 2010; Riemensperger et al., 2011; Cichewicz et al., 2016). The simplest interpretation of the behavioral consequences observed in DA-deficient flies is that the observed phenotypes are solely due to the lack of brain DA. However, quiescence behavior can be induced by reduced DA (Riemensperger et al., 2011; Cichewicz et al., 2016) or increased 5-HT signaling (Pooryasin and Fiala, 2015). Similarly, we have shown that phototactic behavior is strongly decreased in DA deficient flies, whereas increased 5-HT1A receptor signaling has similar effects in honeybees (Thamm et al., 2010). Thus, it seems likely that 5-HT and DA may antagonize each other, with opposing behavioral effects. Similar interactions of dopaminergic and serotoninergic systems occur in the context of arousal in mammals (Wong et al., 1995; Sasaki-Adams and Kelley, 2001; Daw et al., 2002). Consequently, the impact of the 5-HT system in the control of DA neuron activity appears to be a pivotal factor in motor, mood and cognitive effects of DA therapies (reviewed in De Deurwaerdère and Di Giovanni, 2016).

DA neuron denervation strongly alters 5-HT neuron innervation in the rat striatum (Rylander et al., 2010) and increases overall 5-HT IR in the caudate nucleus and globus palidus (Zeng et al., 2010). Moreover, 5-HT transporters in the putamen are significantly increased in PD patients receiving L-DOPA treatment and suffering from LID, as well as in primates developing dyskinesia from L-DOPA treatment (Rylander et al., 2010). As with observations in human patients and in PD vertebrate models (Linazasoro, 2005; Rylander et al., 2010; Zeng et al., 2010) our data show that 5-HT producing neurons respond to DA deficiency with hyper-innervation of some target regions. However, consequences of long-term DA deficiency on the proper development of 5-HT producing neurons cannot be ruled out and a long-term effect may not be directly transferable to PD-like conditions in a brain suffering progressive DA neuron degeneration. Yet even with these different time scales, we see reciprocal effects consistent with competitive interactions between DA and 5-HT.

However, our data show decreases in 5-HT levels in terminal regions and altered 5-HT neuron plasticity in wild type brains with enhanced DA subsequent to L-DOPA treatment. The DPM 5-HT neurons (Lee et al., 2011; Haynes et al., 2015) react to 10 days L-DOPA treatment with decreased 5-HT in their terminal region on the MB vertical lobes, the same region showing enhanced 5-HT subsequent to DA deficiency. This decreased intensity of 5-HT neuron terminals could indicate that pharmacologically increased DA signaling through L-DOPA feeding may have an acute impact on 5-HT neuron plasticity in the fully developed brain and negatively influence outgrowth of 5-HT producing neurons even under wild-type conditions. However, consequences of L-DOPA treatment on 5-HT neuron functionality cannot be excluded.

**Figure 7:**
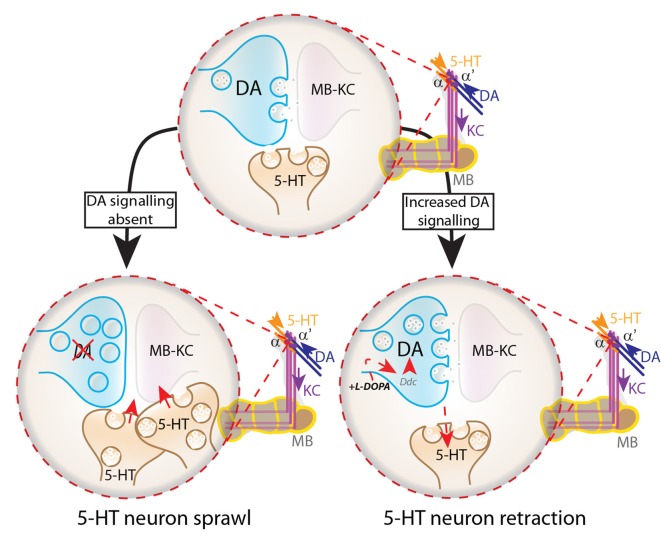
DA modulates serotonin connectivity in the *Drosophila* brain. Under wild-type conditions, dopaminergic neurons predominantly innervate the tips of the α-lobes, whereas 5-HT neurons predominantly innervate the α’-lobes. Lack of DA signaling results in sprawling of 5-HT neurons at the tips of the α-lobes, whereas L-DOPA-induced increase in DA synthesis triggers retraction of 5-HT neurons from their target region on the MB α- and α’-lobes.

In human patients the administration of L-DOPA represents currently the most effective pharmacological treatment for PD, but long-term treatment is hampered by the development of dyskinesia and motor fluctuations, the so-called L-DOPA-induced dyskinesia (LID). The exact cause of LID is unknown, but dysfunctional 5-HT neuron plasticity triggered by the combined effects of DA neuron denervation and pharmacological DA replacement with L-DOPA have been implicated (Calabresi et al., 2000; Hirsch, 2000; Cenci and Lundblad, 2006; Cenci and Lindgren, 2007). Our data indicate that the DA/5-HT competitive interactions can occur in a more normal situation than total DA deficiency (Figure 7). Indeed, these neurons undergo age-dependent plasticity (Tonoki and Davis, 2015). The observed DA/5-HT competitive interactions and the similarities between *Drosophila* and vertebrate models for PD may open novel vistas to better understand the development of LID e.g., through testing how these 5-HT neurons react to L-DOPA treatment under unbalanced DA/5-HT signaling in *d*TH deficient flies, completely devoid of DA signaling in the brain or under neurodegenerative conditions mimicking PD in flies.

## Author Contributions

TDR, JN, FR, BC, KC, JE, Y-TL and KC performed and analyzed experiments. TDR designed and supervised the study. TDR and JH wrote the manuscript.

## Conflict of Interest Statement

The authors declare that the research was conducted in the absence of any commercial or financial relationships that could be construed as a potential conflict of interest.

## Supplementary Material

FIGURE S1 DA-deficient flies show increased 5-HT IR in neurons of the posterior lateral protocerebrum. Quantified 5-HT immune reactivity of neurons in the posterior lateral protocerebrum (PLP) stained with anti-5-HT (rat) in *w*^1118^ and DA-deficient flies. n.s.: *p* > 0.05; **p* < 0.05.
