# Supplementary figures and images for: Dopamine Modulates Serotonin Innervation in the Drosophila Brain
Source: Front Syst Neurosci. 2017 Oct 16;11:76. doi: 10.3389/fnsys.2017.00076 (PMC5650618; doi:10.3389/fnsys.2017.00076)

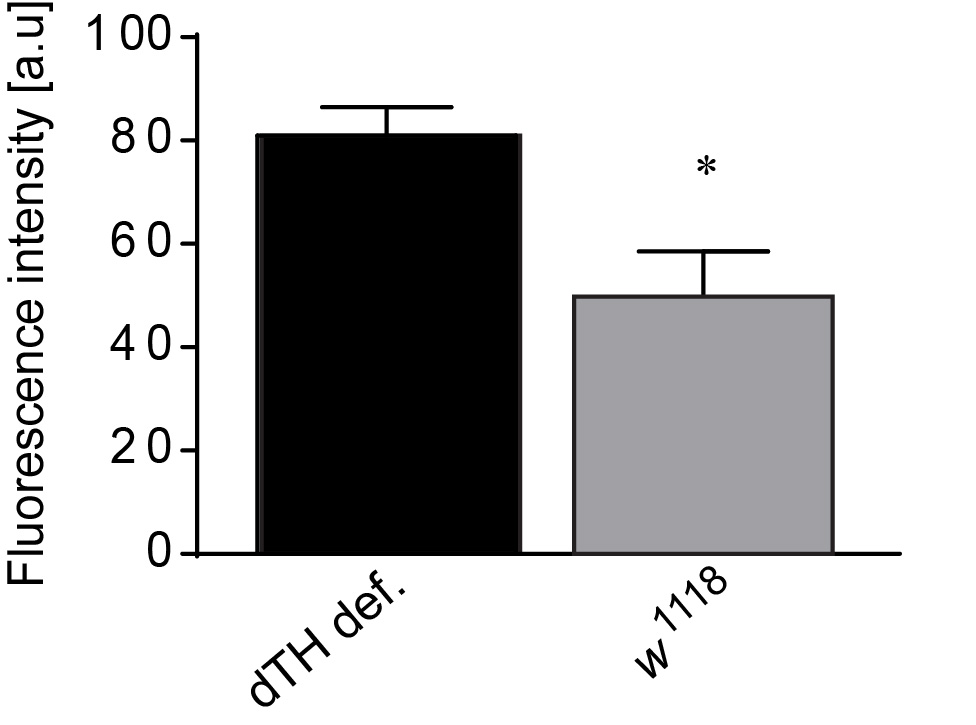

Supplement: FIGURE S1 — DA-deficient flies show increased 5-HT IR in neurons of the posterior lateral protocerebrum. Quantified 5-HT immune reactivity of neurons in the posterior lateral protocerebrum (PLP) stained with anti-5-HT (rat) in w1118 and DA-deficient flies. n.s.: p > 0.05; *p < 0.05. [file Image_1.jpeg]
